# The efficacy and safety of docetaxel with cisplatin compared with other chemotherapies in definitive chemoradiotherapy for head and neck squamous cell carcinoma: a real-world study

**DOI:** 10.1007/s10147-026-02988-2

**Published:** 2026-02-20

**Authors:** Takumi Kumai, Yuto Izumiya, Misaki Hayashi, Kaoru Miyakoshi, Takahiro Inoue, Hisataka Ominato, Motozumi Nakamura, Risa Wakisaka, Daisuke Araki, Michihisa Kono, Hidekiyo Yamaki, Kenzo Ohara, Yoshiya Ishida, Tomoki Yoshizaki, Tetsuji Wada, Nobuyuki Bandoh, Miki Takahara

**Affiliations:** 1 Department of Otolaryngology Head & Neck Surgery, Asahikawa Medical University, Midorigaoka-Higashi 2-1-1-1, Asahikawa, 078-8510 Japan; 2 Department of Innovative Head & Neck Cancer Research and Treatment, Asahikawa Medical University, Asahikawa, Japan; 3 Department of Otolaryngology, Nikko Memorial Hospital, Muroran, Japan; 4 Department of Otolaryngology-Head and Neck Surgery, Hokuto Hospital, Obihiro, Japan; 5 Department of Otolaryngology, Head and Neck Surgery, Kitami Red Cross Hospital, Kitami, Japan; 6 Department of Otolaryngology-Head and Neck Surgery, Asahikawa Kosei Hospital, Asahikawa, Japan

**Keywords:** CCRT, Head and neck, HNSCC, Docetaxel, Cisplatin

## Abstract

**Background:**

Regarding concurrent chemoradiotherapy (CCRT), the combination of docetaxel and cisplatin (DP regimen) is a promising option for treating head and neck squamous cell carcinoma (HNSCC) with a relatively low dose of cisplatin; however, its non-inferiority to other chemotherapies in efficacy and tolerability remains unclear.

**Methods:**

In this retrospective multi-institutional study, the efficacy and safety of the DP regimen were compared with those of other chemotherapy regimens in patients who underwent CCRT for HNSCC. Overall survival, progression-free survival, and adverse effects—including estimated glomerular filtration rate (eGFR)—were evaluated as outcome measures.

**Results:**

Study results showed that a total of 211 patients were included. The prevalence of oropharyngeal, hypopharyngeal, and laryngeal cancer was 44%, 33%, and 17%, respectively. Overall response and recurrence rates were comparable between the DP regimen and high-dose CDDP alone. Although overall and progression-free survival tended to be longer with the DP regimen than with high-dose CDDP, the differences were not statistically significant. Neutropenia was more frequently observed with the DP regimen, but the chemotherapy completion rate was comparable to that of high-dose CDDP alone. Regarding renal function, eGFR significantly decreased with high-dose CDDP but not with the DP regimen.

**Conclusions:**

A combination of docetaxel and cisplatin in concurrent chemoradiotherapy was a favorable option for treating HNSCC with acceptable efficacy and manageable toxicity.

**Supplementary Information:**

The online version contains supplementary material available at 10.1007/s10147-026-02988-2.

## Introduction

Head and neck squamous cell carcinoma (HNSCC) is the sixth most common malignancy worldwide, with more than 900,000 new cases diagnosed annually [1]. Between the 2000s and 2010s, the age-adjusted incidence rate of stage IV HNSCC increased significantly by 26.1% [2]. Concurrent chemoradiotherapy (CCRT) is a feasible treatment option for advanced HNSCC. Because HNSCC cells often harbor chemotherapy-resistant genes [3], radiotherapy is usually combined with chemotherapy to achieve synergistic effects.

The development of chemotherapy regimens for CCRT in HNSCC began in the 1990s. In 1996, Merlano et al. reported that the combination of 5-fluorouracil (5-FU) and cisplatin (CDDP) (FP regimen) had additive effects with radiotherapy, improving complete response and overall survival [4]. Several phase III studies later demonstrated that high-dose CDDP alone could be an alternative to the FP regimen [5, 6], although differences in radiotherapy methods and tumor sites among studies were noted. Since the 2010s, high-dose CDDP alone has been considered a standard regimen for CCRT in HNSCC.

The complete response rate of first-line CCRT in HNSCC is approximately 40–70% [7–9], indicating that second- or third-line treatments are required for patients with recurrence. Immune checkpoint inhibitors and targeted therapies have emerged as promising second-line options. Pembrolizumab, a PD-1 inhibitor, and cetuximab are often combined with the high-dose CDDP-based regimen [10, 11]. To enable the administration of second- or later-line treatments including high-dose CDDP during recurrence, the preservation of renal function is essential. To avoid the nephrotoxicity associated with high-dose CDDP during first-line CCRT [12], we developed the DP regimen—a combination of docetaxel (DOC) and CDDP at a reduced dose (from 100 mg/m^2^ to 60 mg/m^2^) [13]. This regimen was well tolerated and demonstrated favorable efficacy compared with historical controls. In this study, we compared the efficacy and safety of the DP regimen with other regimens, including high-dose CDDP alone, in a multi-institutional retrospective study to further validate the clinical utility of the DP regimen in CCRT.

## Materials and methods

### Patients

A total of 249 newly diagnosed patients with head and neck squamous cell carcinoma (HNSCC) who received first-line concurrent chemoradiotherapy (CCRT) at Asahikawa Medical University, Nikko Memorial Hospital, Hokuto Hospital, Kitami Red Cross Hospital, and Asahikawa Kosei Hospital during January 2019 to December 2023 were retrospectively reviewed. Patients lacking sufficient clinical information were excluded, resulting in 211 eligible cases for this study. Eligibility criteria were as follows: 1. patients aged > 18 years with histologically confirmed HNSCC. 2. Availability of clinical information including TNM classification based on the 8th edition of the Union for International Cancer Control (UICC) staging system and prognostic outcomes. 3. Stage III or IV HNSCC, stage I or II p16-positive oropharyngeal carcinoma, nasopharyngeal carcinoma, or advanced T2 hypopharyngeal carcinoma treated with definitive CCRT. 4. Karnofsky performance status (KPS) ≥ 60% and no prior surgery, radiotherapy, or chemotherapy for HNSCC. 5. No history of other malignancies. Approval for data collection and analysis was obtained from the Institutional Review Board of Asahikawa Medical University (approval no. 20054 and 25,111). Tumors were classified according to the AJCC 8th edition.

### Treatment

All patients underwent definitive CCRT. Because this was a multi-institutional retrospective study, the choice of concurrent chemotherapy regimen was not protocol-mandated and was determined by institutional treatment policies and physician discretion, considering patient-related factors such as renal function, age, and performance status. Both regimens were used during overlapping periods, and treatment selection was not strictly era-dependent. The planned irradiation dose was 60–70 Gy, and more than 85% of patients received intensity-modulated radiation therapy (IMRT). DP regimen: DOC (50 mg/m^2^, day 1) and CDDP (15 mg/m^2^/day, days 2–5) were administered every 3 weeks and continued for at least two cycles (Supplementary Fig. 1) [13]. CDDP regimen: CDDP (100 mg/m^2^, day 1) was administered every 3 weeks for at least two cycles [6]. CBDCA regimen: carboplatin (CBDCA, AUC = 5, day 1) was administered every 3 weeks for at least two cycles [14]. TPF regimen: DOC (50 mg/m^2^, day 1), CDDP (60 mg/m^2^, day 4), and 5-FU (600 mg/m^2^, days 1–5) were administered every 3 weeks for at least two cycles [15]. TPS regimen: DOC (60 mg/m^2^, day 8), CDDP (60 mg/m^2^, day 8), and S-1 (80 mg/m^2^/day, twice daily on days 1–14) were administered every 4 weeks for at least two cycles [16]. For patients considered unsuitable for CDDP-based chemotherapy, CDCAE (AUC = 5, day 8) was administered (TCS regimen). DOC regimen: DOC (20 mg/body, day 1) was administered weekly for at least four cycles [17].

### Response and toxicity

Tumor response was evaluated 8–12 weeks after completion of therapy using the Response Evaluation Criteria in Solid Tumors (RECIST) version 1.1. Responses were categorized as complete response (CR), partial response (PR), stable disease (SD), or progressive disease (PD). Overall survival (OS) was defined as the time from the start of radiotherapy to death from any cause. PFS was defined as the time from the start of radiotherapy to disease progression or death from any cause, whichever occurred first. Disease-specific survival is defined as the interval from treatment initiation to death directly attributable to the underlying disease, while deaths from unrelated causes are censored from the analysis. Adverse events more than grade 3 according to the Common Terminology Criteria for Adverse Events (CTCAE) version 5.0 were listed. Completion of chemotherapy was defined as administration of at least two cycles without switching regimens. For weekly docetaxel-based regimens, chemotherapy completion was defined as receiving 4 planned weekly doses. Completion of radiotherapy was defined as delivery of the planned total radiation dose. Estimated glomerular filtration rate (eGFR) was calculated using the following formula: eGFR = 194 × serum creatinine − 1.094 × age − 0.287 × (0.739 if female) [18].

### Ethical consideration

This study was conducted in accordance with the Declaration of Helsinki. The protocol was approved by the Institutional Review Board of Asahikawa Medical University (approval no. 20054 and 25,111). Informed consent was obtained using an opt-out procedure.

### Statistical analysis

Overall and progression-free survival curves were estimated using the Kaplan–Meier method, and differences were analyzed using the log-rank test. Clinical parameters and renal function after treatment were compared using the Chi-square test or Student’s *t*-test. A *p*-value < 0.05 was considered statistically significant. All statistical analyses were performed using Prism 9 (ver. 9.4.1).

## Results

### Patient characteristics

A total of 249 patients were recruited, and 211 eligible patients were included in this study. The inclusion criteria are listed in the *Materials and methods* section. Patient characteristics are summarized in Table 1. Eighty-five percent of the patients were male, and the median age was 70 years (range, 31–91 years). Alcohol consumption and smoking were observed in 86% and 76% of patients, respectively. The median Brinkman index was 800 (range, 100–2880). Forty-four percent of patients had oropharyngeal cancer, among which 67% were p16 positive. The second most common primary site was the hypopharynx (33%), followed by the larynx (17%). Eight patients had nasopharyngeal cancer, three had oral cancer, and one had sinonasal cancer. According to the staging system for p16-positive oropharyngeal cancer, 39% and 61% of patients were classified as Stage I/II and Stage III/IV, respectively.

**Table 1:** Patients characteristics

Sex (male: female)		180:31
Age (median)		31–91 (70)
Drinking behavior		182 (86%)
Current smoker		161 (76%)
Brinkman Index (median)		100–2880 (800)
Location of tumor	Oropharynx (total)	93 (44%)
	Oropharynx (p16 +)	62 (67% of oropharynx))
	Hypopharynx	70 (33%)
	Larynx	36 (17%)
	Others	12 (6%)
Stage	I	34 (16%)
	II	49 (23%)
	III	38 (18%)
	IV	90 (43%)
Best objective response	CR	180 (85%)
	PR	26 (12%)
	SD	4 (2%)
	PD	1 (1%)
Recurrence	Total	66 (31%)
	Local	21 (9%)
	Nodal	27 (13%)
	Distant metastasis	27 (13%)
Clinical outcome	Disease-specific	28 (13%)
	death	
	Non-disease-specific death	14 (7%)

The median follow-up period was 35 months (range, 2–75 months). The overall response rate to CCRT was 97%, with complete response (CR) and partial response (PR) observed in 85% and 12% of patients, respectively. Recurrence occurred in 31% of patients. Local, nodal, and distant recurrences were observed in 9%, 13%, and 13% of patients, respectively. During the follow-up period, disease-specific death occurred in 13% of patients, while non-disease-specific death occurred in 7%.

### Clinical responses to CCRT in patients with HNSCC

**Fig. 1:**
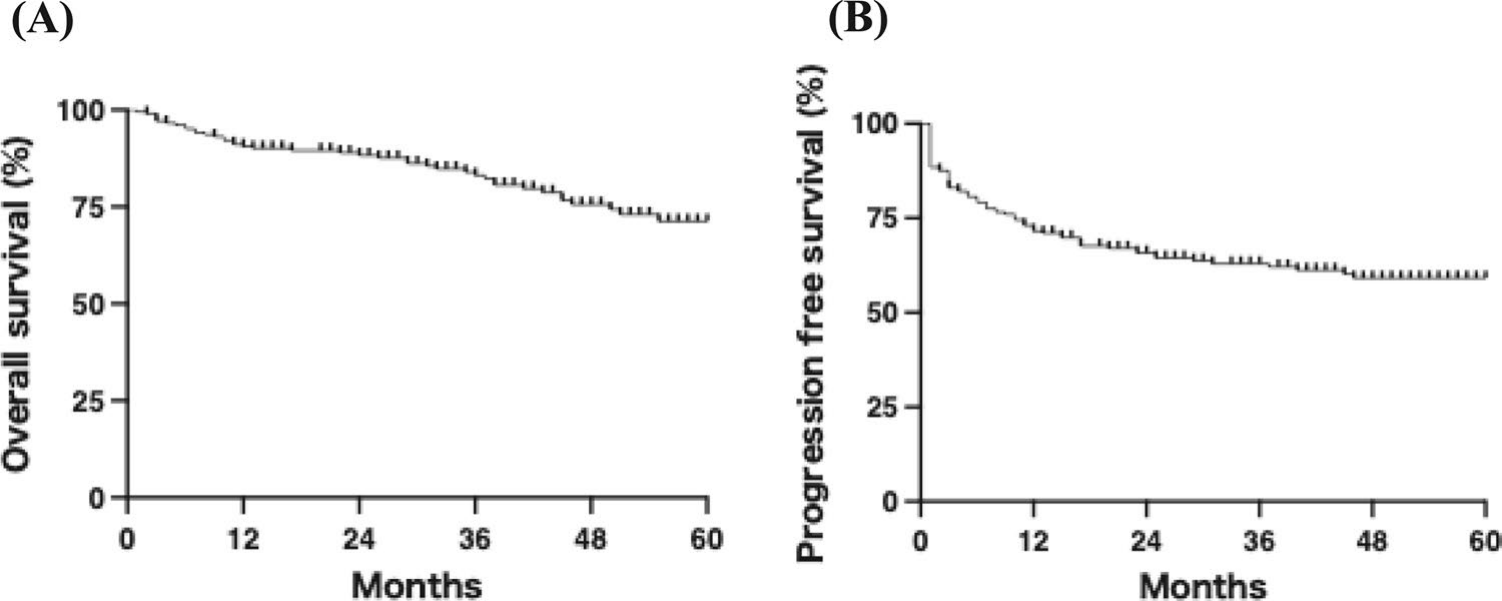
Overall and progression-free survival rates in all patients. **A** The 1-year and 5-year overall survival rates were 91% and 72%, respectively. **B** The 1-year and 5-year progression-free survival rates were 74% and 60%, respectively

**Fig. 2:**
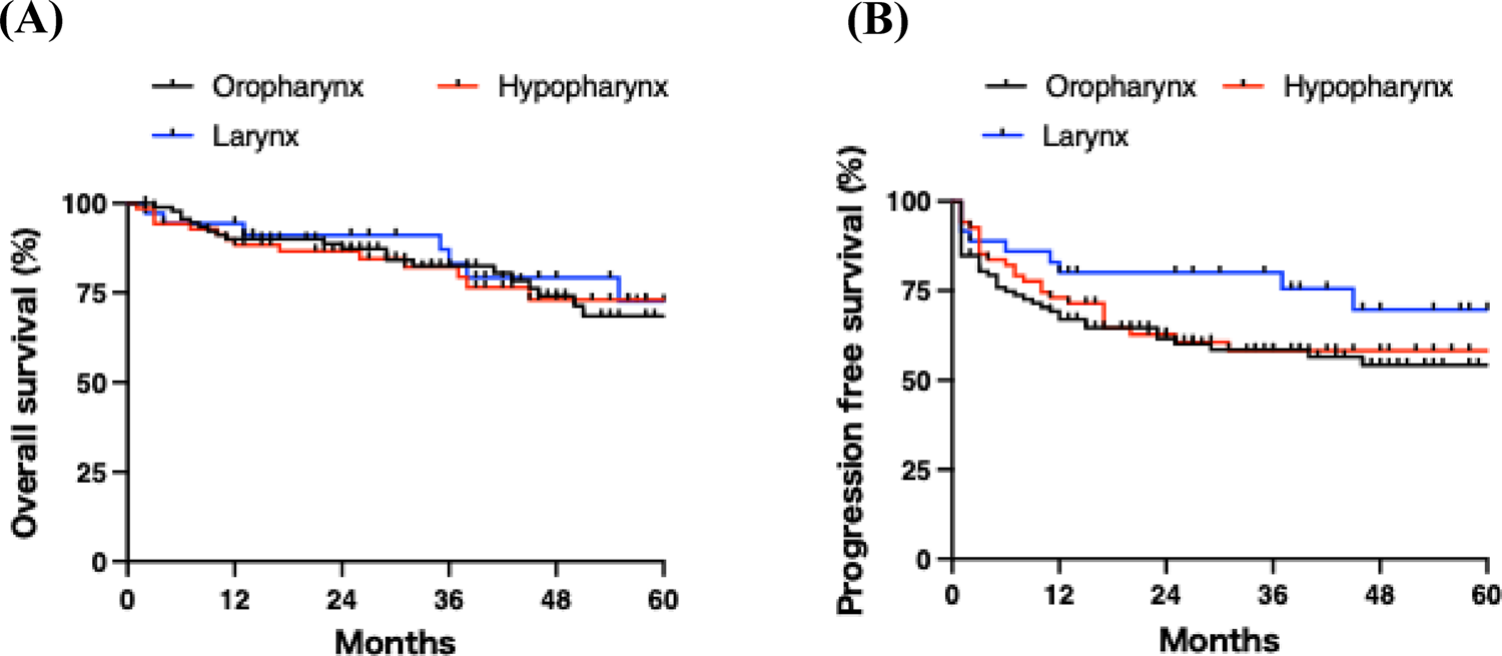
Overall and progression-free survival rates stratified by primary site. **A** Overall and **B** progression-free survival curves are shown according to primary lesion site. No significant differences were observed among the primary sites

**Fig. 3:**
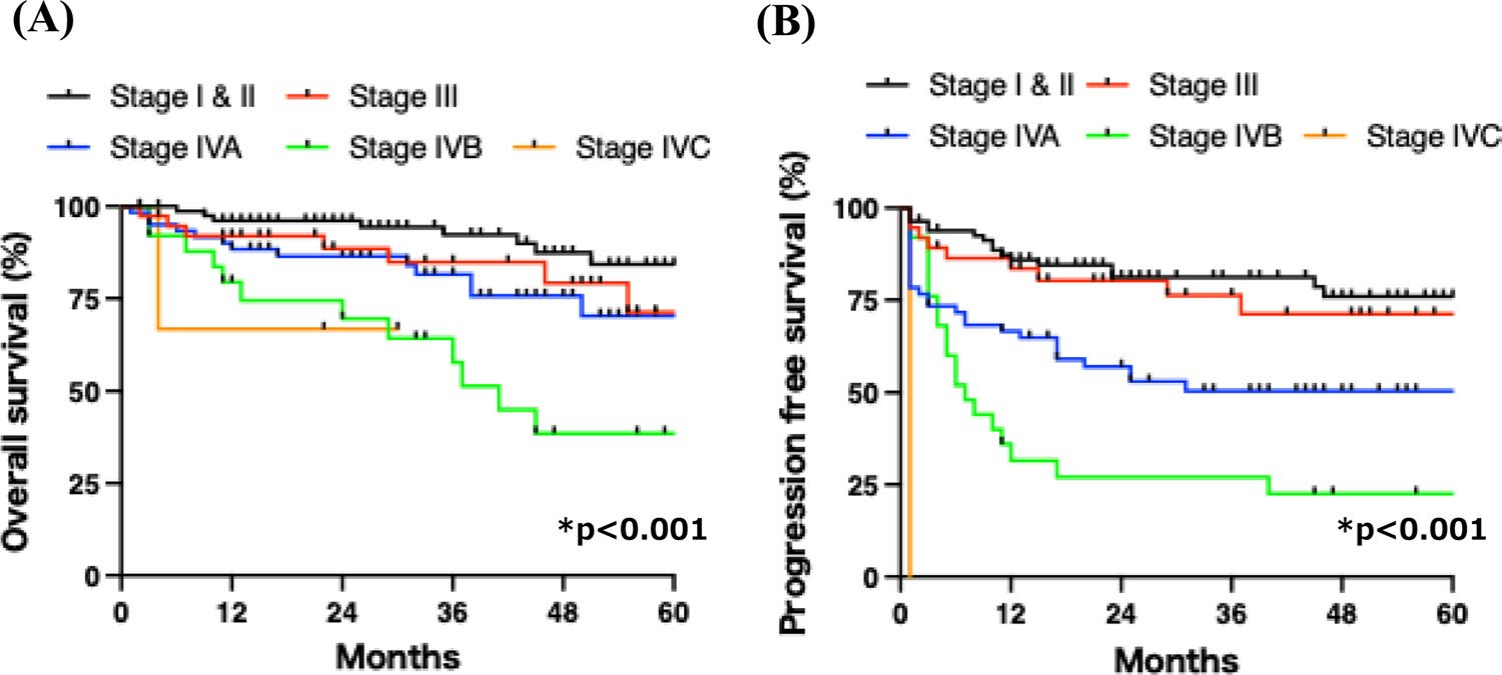
Overall and progression-free survival rates according to clinical stage. **A** Overall and **B** progression-free survival curves are shown according to clinical stage. Survival rates significantly decreased with higher stage

Next, we evaluated the survival outcomes of patients with HNSCC treated with CCRT. As shown in Fig. 1A, the 1-year and 3-year OS rates were 91% and 83%, respectively, with a median OS of 71 months. The 1-year and 3-year PFS rates were 71% and 63%, respectively (Fig. 1B). OS did not differ significantly among the primary tumor sites (Fig. 2A). Although PFS tended to be more favorable in laryngeal cancer, the difference was not statistically significant (Fig. 2B; p = 0.1). As expected, p16 positivity was significantly associated with improved OS and PFS in oropharyngeal cancer (Supplementary Fig. 2A and B). Higher clinical stage was associated with poorer OS and PFS (Fig. 3A and B), suggesting that the study population was appropriate and representative for the analysis.

### The efficacy of DP and other regimens in CCRT against HNSCC

We next evaluated the efficacy of each chemotherapy regimen. Forty-three percent and 27% of patients received DP and CDDP regimens, respectively (Table 2). Twenty-one percent of patients received a triple combination regimen such as TPF. The chemotherapy completion rate exceeded 90% for all regimens except the triple combination regimen. The radiotherapy completion rate was over 95% in the top three regimens. The overall response rates were 100%, 95%, and 100% in the DP, CDDP, and triple combination regimens, respectively. Recurrence rates were higher in the triple combination and CBDCA regimens, whereas recurrence rates were comparable between the DP and CDDP regimens. Eligibility for second-line treatment among patients with recurrence was comparable across regimens except for CBDCA.

**Table 2:** The clinical outcomes between chemotherapy regimens

	*n*	Chemotherapy completion rate	Radiotherapy completion rate	Overall response rate	Recurrence rate	Eligibility for 2nd line treatment
DP	91	87 (96%)	91 (100%)	91 (100%)	22 (24%)	84 (92%)
CDDP	56	51 (91%)	53 (95%)	53 (95%)	16 (29%)	56 (100%)
TPF, TPS or TCS	45	40 (89%)	44 (98%)	45 (100%)	22 (49%)	41 (91%)
DOC	11	11 (100%)	10 (91%)	10 (91%)	2 (19%)	11 (100%)
CBDCA	8	8 (100%)	8 (100%)	7 (88%)	4 (50%)	6 (75%)
Total	211					

*DP* Cisplatin + docetaxel, *DSS* Disease-specific survival rate, *TCS* Carboplatin + docetaxel + S-1, *TPF* Cisplatin + docetaxel + 5-FU, *TPS* Cisplatin + docetaxel + S-1

^*^The TPF, TPS, and TCS regimens were analyzed as a single group owing to the limited sample size of each regimen, which precluded separate statistical analyses

**Fig. 4:**
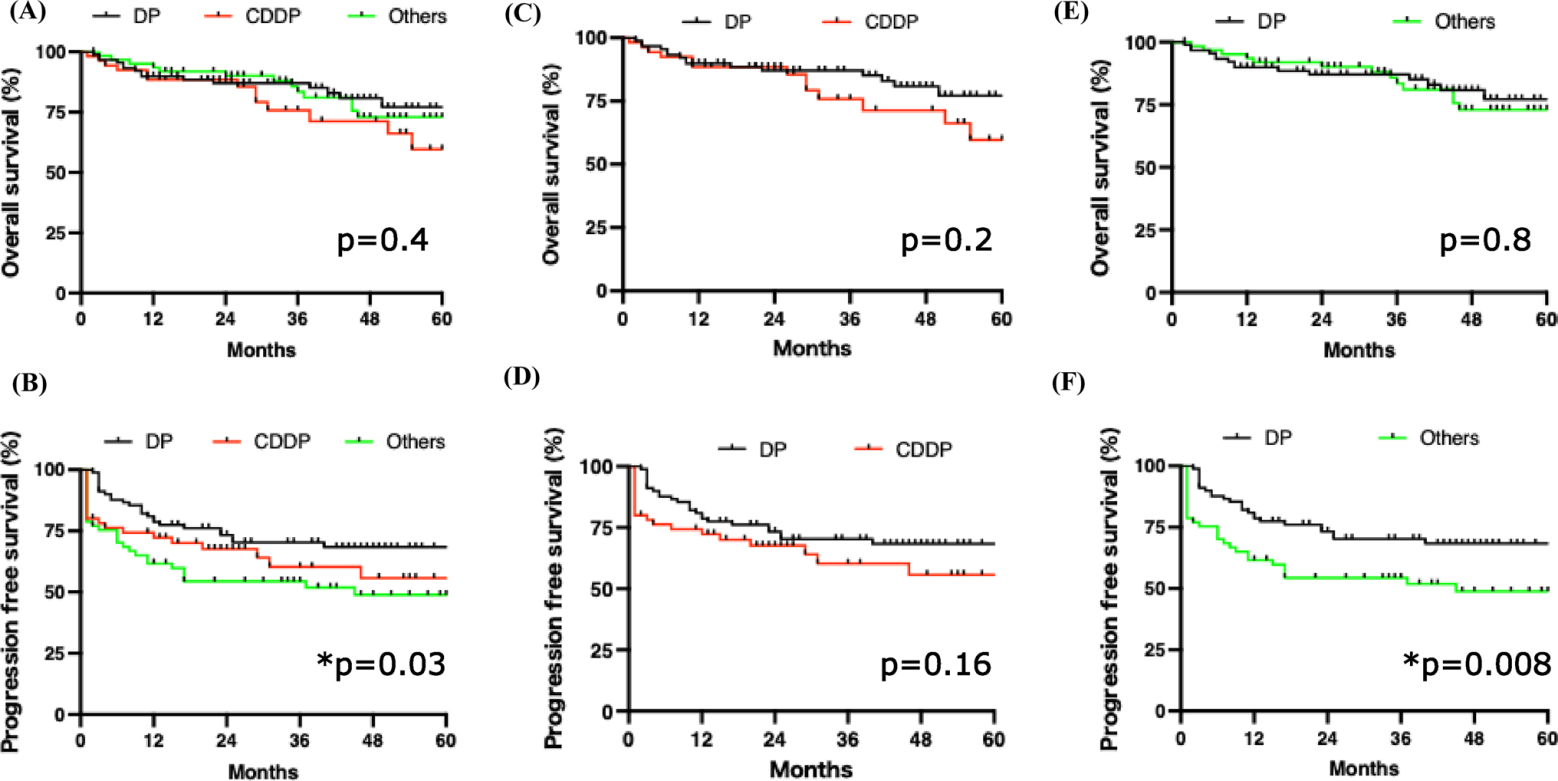
Overall and progression-free survival rates according to chemotherapy regimen. **A** Overall and **B** progression-free survival rates in patients treated with DP, CDDP, or other regimens. **C** Overall and **D** progression-free survival rates in patients treated with DP or CDDP regimens. **E** Overall and **F** progression-free survival rates in patients treated with DP or regimens other than DP or CDDP

Next, we compared survival outcomes among the different chemotherapy regimens. As shown in Fig. 4A, OS rates were comparable between DP and non-DP regimens. Interestingly, the PFS rate was more favorable in the DP regimen compared with non-DP regimens (Fig. 4B). Given that disease-specific survival closely approximated OS due to the rarity of non-disease-specific deaths (Supplementary Fig. 3), subsequent analyses focused on OS and PFS. Although both OS and PFS tended to be better in the DP regimen than in the CDDP regimen, the differences were not statistically significant (Fig. 4C and D). PFS was significantly improved in patients receiving the DP regimen compared with those treated with regimens other than DP or CDDP (Fig. 4E and F). Within the analysis stratified by tumor subsite, the DP regimen yielded significantly better PFS than other regimens in hypopharyngeal cancer (Supplementary Fig. 4). In oropharyngeal cancer, OS and PFS were comparable across chemotherapy regimens, regardless of p16 positivity.

### Adverse effects of DP and other regimens in CCRT

**Fig. 5:**
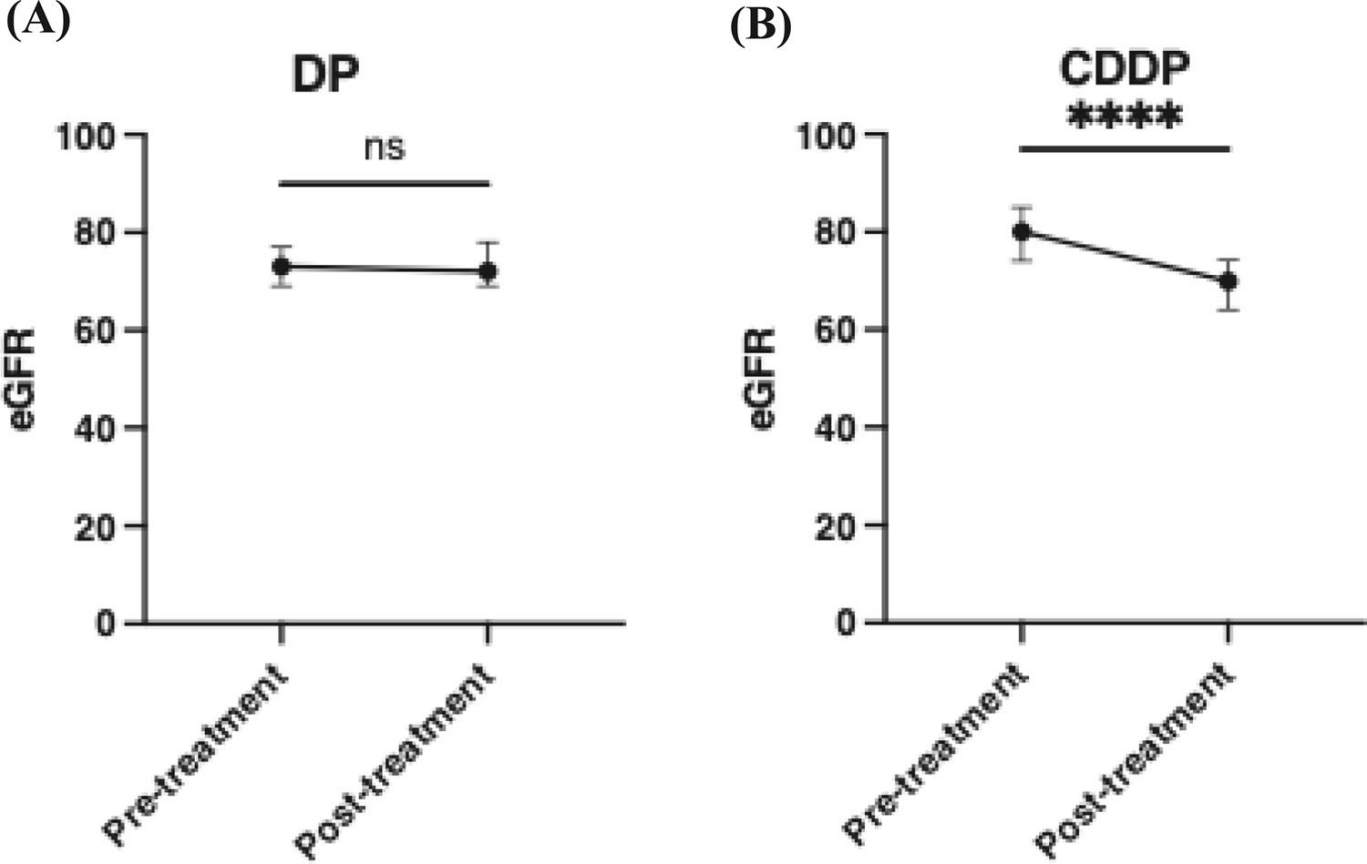
Renal function before and after chemoradiotherapy with DP or CDDP regimens. **A** and **B** Estimated glomerular filtration rate (eGFR) before and after chemoradiotherapy with DP or CDDP regimens, respectively. eGFR was maintained with the DP regimen but decreased with the CDDP regimen. ****p < 0.001

Adverse events of grade 3 or higher according to CTCAE ver. 5.0 were evaluated for each chemotherapy regimen. Neutropenia, which occurred more frequently in patients receiving the DP or triple combination regimen, was manageable, as evidenced by acceptable completion rates of both chemotherapy and radiotherapy (Table 3). Similarly, mucositis was more common in the DP group but was clinically manageable, as reflected by comparable treatment completion rates. Anorexia was observed in 20% of patients treated with the CDDP regimen. Grade 3 renal dysfunction occurred exclusively in the CDDP group. As shown in Fig. 5, eGFR remained comparable before and after treatment in patients receiving the DP regimen, whereas it significantly decreased in those treated with the CDDP regimen, suggesting that the DP regimen may be an effective and well-tolerated chemotherapy option capable of preserving renal function.

**Table 3:** The adverse effects of chemotherapy more than grade 3 in CTCAE ver5.0

	*n*	Neutro-penia	Anemia	Thrombo-cytopenia	Liver dysfunction	Renal dysfunction	Hypo- natremia	Mucositis	Anorexia	Diarrhea
DP	91	76 (84%)	5 (5%)	2 (2%)	1 (1%)	0 (0%)	2 (2%)	41 (45%)	4 (4%)	0 (0%)
CDDP	56	12 (21%)	1 (2%)	1 (2%)	0 (0%)	1 (2%)	1 (2%)	11 (20%)	11 (20%)	0 (0%)
TPF, TPS or TCS	45	42 (93%)	0 (0%)	0 (0%)	0 (0%)	0 (0%)	0 (0%)	3 (7%)	1 (2%)	1 (2%)
DOC	11	0 (0%)	0 (0%)	0 (0%)	0 (0%)	0 (0%)	0 (0%)	0 (0%)	0 (0%)	0 (0%)
CBDCA	8	5 (63%)	0 (0%)	0 (0%)	0 (0%)	0 (0%)	0 (0%)	1 (13%)	0 (0%)	0 (0%)
Total	211									

*DP* Cisplatin + docetaxel, *TCS* Carboplatin + docetaxel + S-1, *TPF* Cisplatin + docetaxel + 5-FU, *TPS* Cisplatin + docetaxel + S-1

^*^The TPF, TPS, and TCS regimens were analyzed as a single group owing to the limited sample size of each regimen, which precluded separate statistical analyses

## Discussion

In this study, we observed that the best response rate, OS, and PFS were comparable between the DP regimen and high-dose CDDP alone in CCRT for HNSCC. The completion rates of chemotherapy and radiotherapy in the DP regimen exceeded 90%, and the overall response rate reached 100%. These values are remarkably high for a real-world study, in which the inclusion criteria were relatively broader than those of phase III trials. Notably, 27% of the patients were older than 75 years, suggesting that the DP regimen is highly tolerable even in elderly individuals. The recurrence rates in patients treated with the DP regimen and high-dose CDDP alone were 24% and 29%, respectively. Among the recurrent cases in the DP group, eligibility for second- or later-line treatment exceeded 90%, indicating that the patients’ general condition was well maintained under this chemotherapy.

Based on phase III trials, high-dose CDDP (100 mg/m^2^ per course) with a cumulative dose exceeding 200 mg/m^2^ is considered the standard regimen for CCRT in the treatment of HNSCC [19, 20]. Although phase III trials demonstrated the efficacy of high-dose CDDP under strict inclusion criteria, real-world evidence indicates that the tolerability of this regimen is relatively poor [21, 22]. Al-Mamgani et al*. l* reported that fewer than 60% of patients with HNSCC could complete the planned courses of high-dose CDDP [20]. This poor compliance is likely attributable to renal dysfunction. Cisplatin accumulation in renal tubular cells induces nephrotoxicity through oxidative and endoplasmic reticulum stress [23]. Cooper et al. further reported that nearly 20% of patients experienced renal toxicity associated with high-dose CDDP during long-term follow-up [24]. In the present study, we also observed a significant decline in eGFR after high-dose CDDP treatment. In the DP group, the cumulative CDDP dose ranged from 60 to 180 mg (median, 120 mg), whereas in the CDDP group, it ranged from 70 to 300 mg (median, 240 mg). The cumulative CDDP dose was significantly higher in the CDDP group (p < 0.001), suggesting that a higher total CDDP exposure may contribute to the development of renal dysfunction. In addition to acute kidney injury, chronic renal failure is frequently observed in patients receiving CDDP-based chemotherapy [23]. Although the risk of progression to end-stage renal disease requiring hemodialysis is low [25], the reduction in eGFR induced by CDDP is generally irreversible. Thus, chronic renal dysfunction caused by high-dose CDDP may limit eligibility for second- or later-line therapies [26].

High-dose cisplatin (> 40 mg/m^2^/day) is associated with renal dysfunction [27]. Therefore, dose reduction of cisplatin is warranted to preserve renal function and to maintain eligibility for second- or later-line chemotherapy. To achieve acceptable oncologic outcomes comparable to those obtained with a cumulative CDDP dose exceeding 200 mg/m^2^, combining cisplatin with other cytotoxic agents may represent a promising strategy for reducing cisplatin exposure. Although the efficacy of high-dose CDDP is well established, no direct comparison between high-dose CDDP and combination regimens with matched patient characteristics or radiotherapy modalities has been reported. In the 1990s and 2000s, 5-FU was commonly combined with CDDP in CCRT, but subsequent studies showed that CDDP alone was associated with fewer adverse events and better survival than CDDP plus 5-FU [28]. DOC is often used to enhance the efficacy of CDDP combined with 5-FU in HNSCC [29]. Furthermore, radiotherapy combined with DOC improves the survival of patients with HNSCC who are ineligible for CDDP compared with radiotherapy alone [17]. Induction of G0/G1 and G2/M phase arrest has been proposed as a major mechanism of DOC as a radiosensitizer [30]. The radiosensitizing effect of DOC can be achieved at extremely low, non-cytotoxic concentrations (< 1 nM), indicating that even a low dose of DOC may be sufficient for concurrent use with radiotherapy. We previously reported that DOC combined with low-dose CDDP (DP regimen) in CCRT demonstrated acceptable efficacy and tolerability in patients with HNSCC [13]. In this study, we provide further evidence that the DP regimen is an effective and renal-sparing alternative to high-dose CDDP.

There have been few reports evaluating the efficacy of adding DOC to CDDP-based CCRT in HNSCC. Inohara et al. conducted phase I and II studies assessing the efficacy and safety of weekly low-dose DOC and CDDP administered concurrently with conventionally fractionated radiotherapy in patients with stage III–IV HNSCC [31, 32]. The primary tumor sites, including the oropharynx, hypopharynx, and larynx, were comparable to those in our cohort. They concluded that this combination therapy achieved satisfactory local control and survival outcomes. Although acute toxicities were common, as observed in our study, late toxicities were negligible. This regimen was also tested in nasopharyngeal carcinoma, showing acceptable toxicity and promising efficacy despite frequent neutropenia during treatment [7]. Beyond HNSCC, the DP regimen has been investigated in several other malignancies. In non-small-cell lung cancer, DP combined with radiotherapy demonstrated favorable survival outcomes with an acceptable safety profile [33]. Similarly, in a randomized controlled trial for cervical cancer, the addition of DOC to CDDP-based radiotherapy tended to improve recurrence-free survival, although the incidence of acute adverse effects was higher; these were reversible, and no increase in late toxicity was observed [34]. DOC with CDDP has also been reported as an effective and well-tolerated regimen in CCRT for esophageal cancer [35]. Collectively, the combination of DOC and CDDP represents an effective CCRT regimen with acceptable toxicity.

This study has several limitations. First, as this was a multi-institutional retrospective study, treatment selection was influenced by institutional practice patterns and physician discretion rather than a uniform protocol. Although both regimens were used during overlapping periods, such inter-institutional variability represents a limitation of the present study and should be considered when interpreting the results. To minimize potential selection bias, we are currently planning a prospective trial comparing the DP regimen with high-dose cisplatin in CCRT for HNSCC. Second, the efficacy of second- or later-line treatments was beyond the scope of the present study. Future trials should therefore evaluate oncological outcomes and adverse events, including renal function, during subsequent treatments in patients initially treated with the DP regimen or other chemotherapy protocols.

With regard to adverse events, the incidence of neutropenia and mucositis was significantly higher in the DP regimen group. However, because the chemotherapy completion rate was comparable between the DP regimen and other regimens, DP-associated neutropenia and mucositis appear to be clinically manageable. Given that strict monitoring of neutrophil counts, prompt administration of granulocyte colony-stimulating factor, and appropriate infection control are essential for neutropenia management, the safety profile of the DP regimen warrants further validation in prospective clinical studies.

## Conclusions

Our study suggests that the DP regimen is associated with acceptable efficacy and tolerability as a CCRT option for patients with HNSCC and may represent an alternative to CDDP monotherapy. The DP regimen showed superior efficacy compared with non-DP regimens, with acceptable safety profiles. Because renal function impairment was negligible with the DP regimen, this chemotherapy could serve as a feasible option to avoid limitations in therapeutic choices caused by renal dysfunction during recurrence.

## Supplementary Information

Below is the link to the electronic supplementary material.

Supplementary file1 (PPTX 340 KB) Supplementary Table 1. The clinical characteristics of patients treated with DP or CDDP regimens. The clinical parameters were compared using the Chi-square test or Student’s t-test. Supplementary Fig. 1. Summary of the DP regimen. Docetaxel (50 mg/m^2^) and cisplatin (15 mg/m^2^/day; total 60 mg/m^2^ per course) were administered on day 1 and days 2–5, respectively. Chemotherapy cycles were repeated every 21 days, and two or three cycles were administered during definitive chemoradiotherapy. Supplementary Fig. 2. Overall and progression-free survival in oropharyngeal cancer. (A) Overall and (B) progression-free survival curves of patients with oropharyngeal cancer stratified by p16 expression. p16 positivity was significantly associated with favorable prognosis. Supplementary Fig. 3. Disease-specific survival rates according to chemotherapy regimen. Disease-specific survival rates in patients treated with DP, CDDP, or other regimens. Supplementary Fig. 4. Overall and progression-free survival rates according to tumor subsite. Overall survival and progression-free survival in patients with p16-positive oropharyngeal cancer (A, B), p16-negative oropharyngeal cancer (C, D), hypopharyngeal cancer (E, F), and laryngeal cancer (G, H)

## Data Availability

All data relevant to the study are included in the article or uploaded as supplementary information.
