# Supplementary material for: The efficacy and safety of docetaxel with cisplatin compared with other chemotherapies in definitive chemoradiotherapy for head and neck squamous cell carcinoma: a real-world study
Source: Int J Clin Oncol. 2026 Feb 20;31(4):730–9. doi: 10.1007/s10147-026-02988-2 (PMC13018028; doi:10.1007/s10147-026-02988-2)
Supplement: Supplementary file 1 — Supplementary file1 (PPTX 340 KB) Supplementary Table 1. The clinical characteristics of patients treated with DP or CDDP regimens. The clinical parameters were compared using the Chi-square test or Student’s t-test. Supplementary Fig. 1. Summary of the DP regimen. Docetaxel (50 mg/m2) and cisplatin (15 mg/m2/day; total 60 mg/m2 per course) were administered on day 1 and days 2–5, respectively. Chemotherapy cycles were repeated every 21 days, and two or three cycles were administered during definitive chemoradiotherapy. Supplementary Fig. 2. Overall and progression-free survival in oropharyngeal cancer. (A) Overall and (B) progression-free survival curves of patients with oropharyngeal cancer stratified by p16 expression. p16 positivity was significantly associated with favorable prognosis. Supplementary Fig. 3. Disease-specific survival rates according to chemotherapy regimen. Disease-specific survival rates in patients treated with DP, CDDP, or other regimens. Supplementary Fig. 4. Overall and progression-free survival rates according to tumor subsite. Overall survival and progression-free survival in patients with p16-positive oropharyngeal cancer (A, B), p16-negative oropharyngeal cancer (C, D), hypopharyngeal cancer (E, F), and laryngeal cancer (G, H) [file 10147_2026_2988_MOESM1_ESM.pptx]

## Slide 1
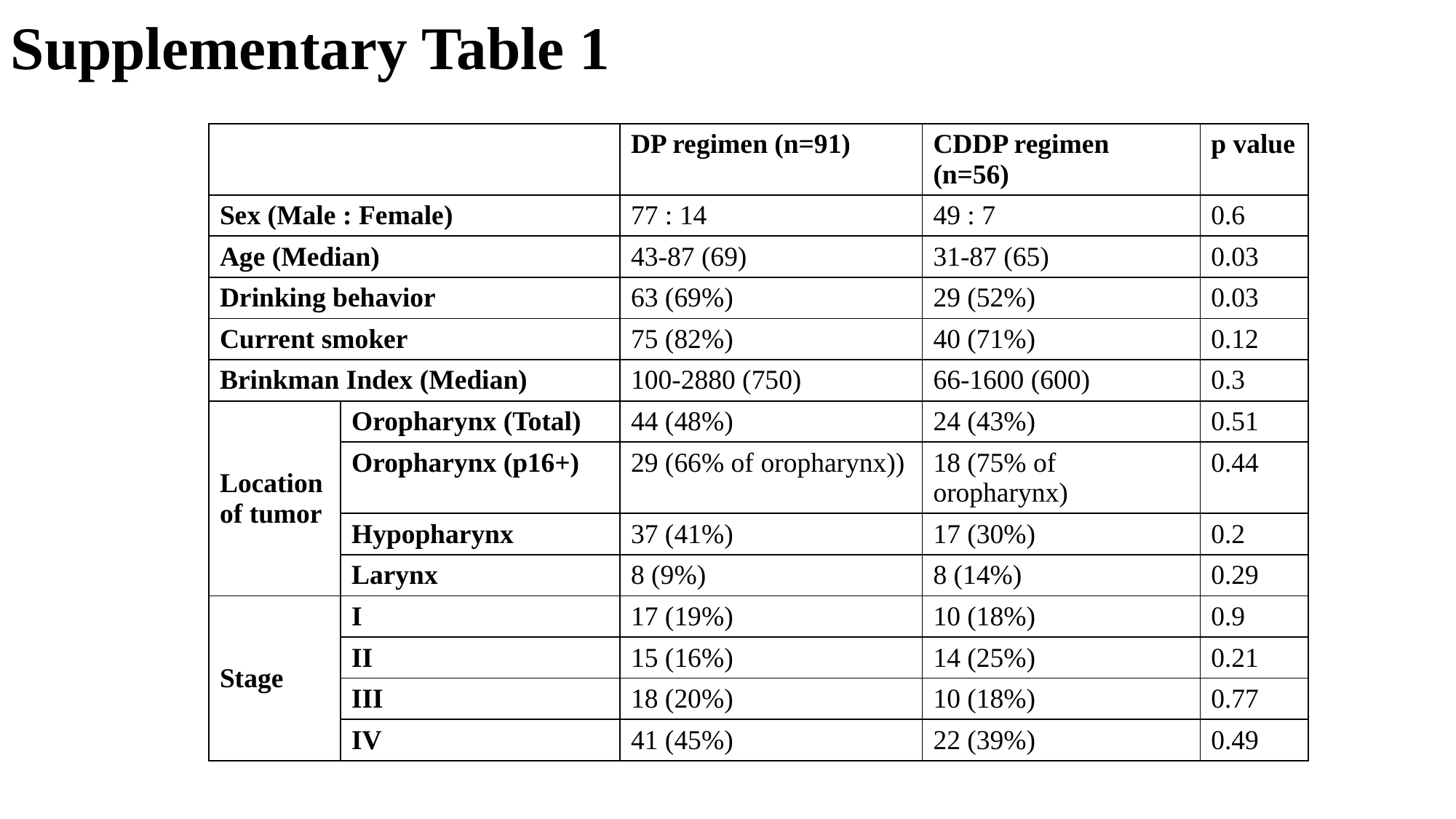

Supplementary Table 1
| | | DP regimen (n=91) | CDDP regimen (n=56) | p value |
| --- | --- | --- | --- | --- |
| Sex (Male : Female) | | 77 : 14 | 49 : 7 | 0.6 |
| Age (Median) | | 43-87 (69) | 31-87 (65) | 0.03 |
| Drinking behavior | | 63 (69%) | 29 (52%) | 0.03 |
| Current smoker | | 75 (82%) | 40 (71%) | 0.12 |
| Brinkman Index (Median) | | 100-2880 (750) | 66-1600 (600) | 0.3 |
| Location of tumor | Oropharynx (Total) | 44 (48%) | 24 (43%) | 0.51 |
| | Oropharynx (p16+) | 29 (66% of oropharynx)) | 18 (75% of oropharynx) | 0.44 |
| | Hypopharynx | 37 (41%) | 17 (30%) | 0.2 |
| | Larynx | 8 (9%) | 8 (14%) | 0.29 |
| Stage | I | 17 (19%) | 10 (18%) | 0.9 |
| | II | 15 (16%) | 14 (25%) | 0.21 |
| | III | 18 (20%) | 10 (18%) | 0.77 |
| | IV | 41 (45%) | 22 (39%) | 0.49 |

## Slide 2
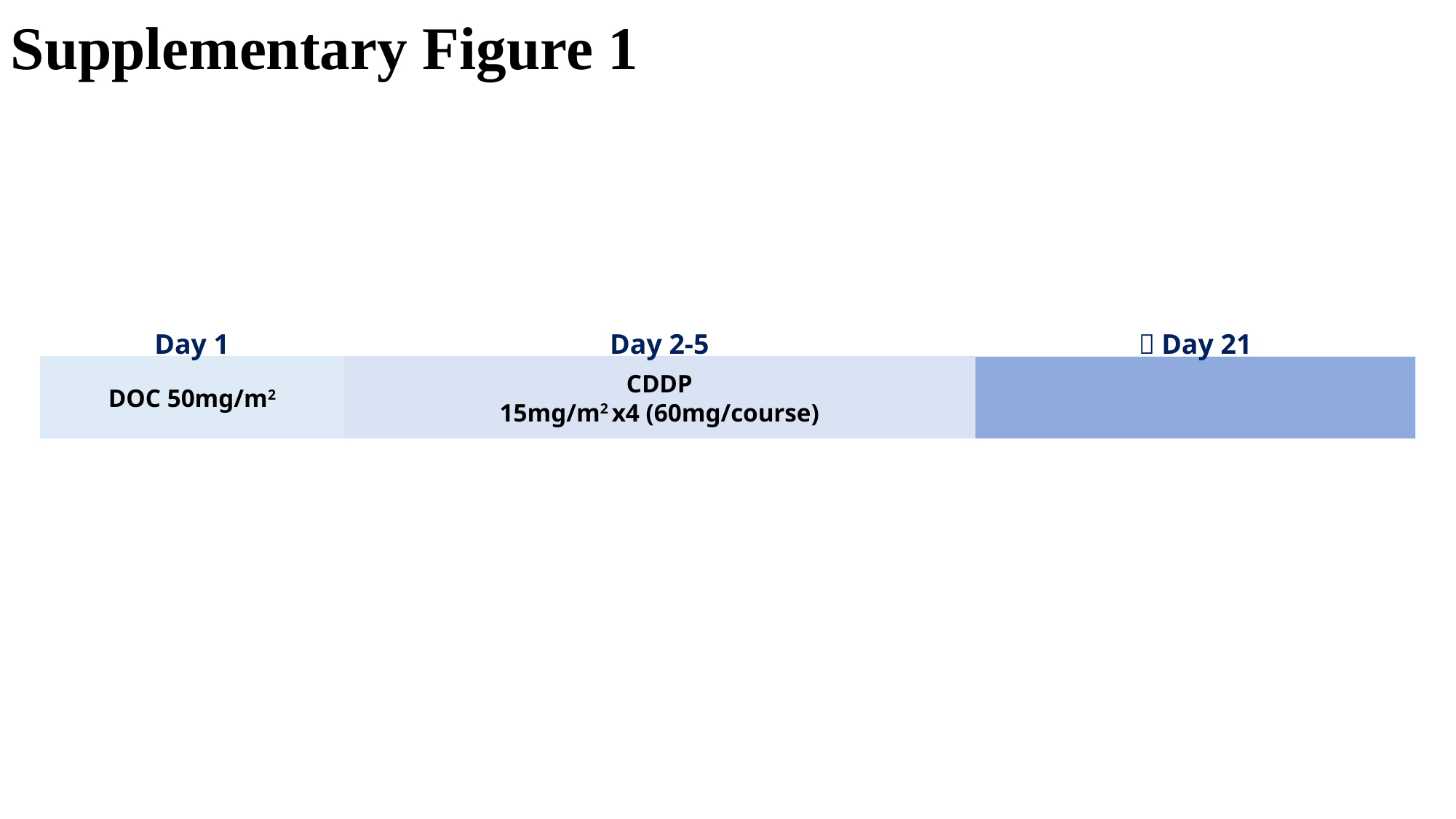

Supplementary Figure 1
Day 1
Day 2-5
〜Day 21
DOC 50mg/m2
CDDP
15mg/m2 x4 (60mg/course)

## Slide 3
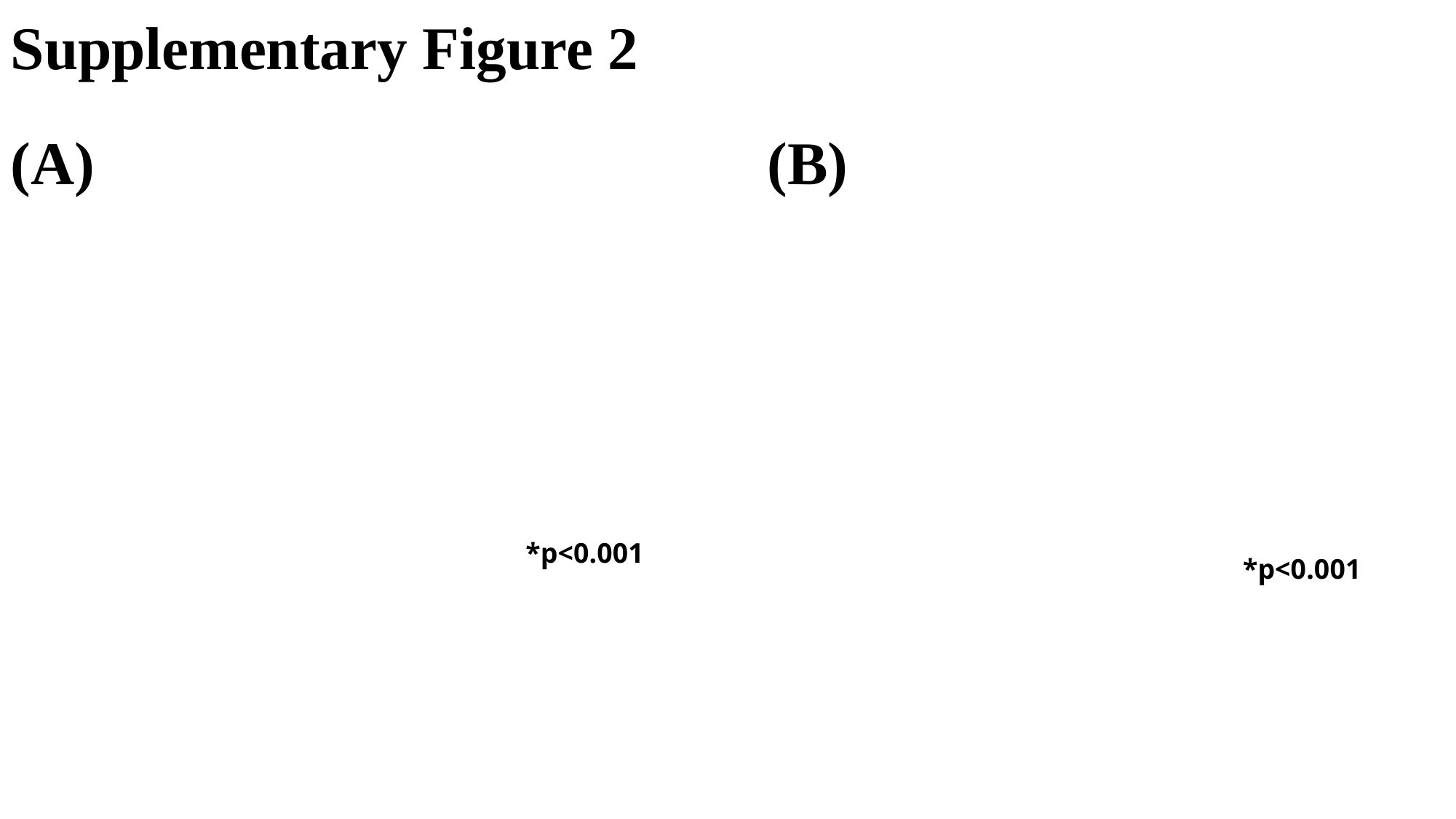

Supplementary Figure 2
(A)
(B)
*p<0.001
*p<0.001

## Slide 4
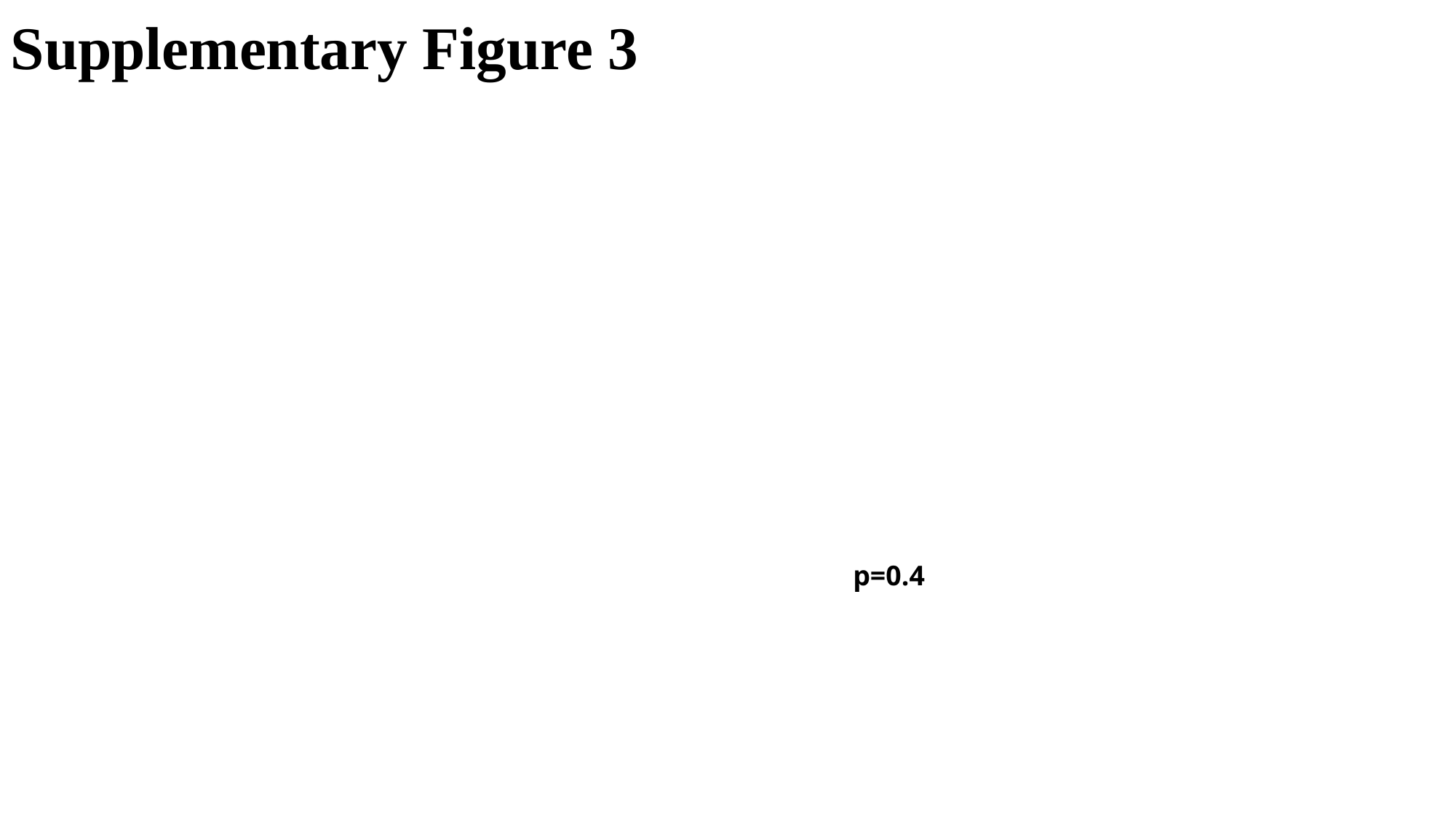

Supplementary Figure 3
p=0.4

## Slide 5
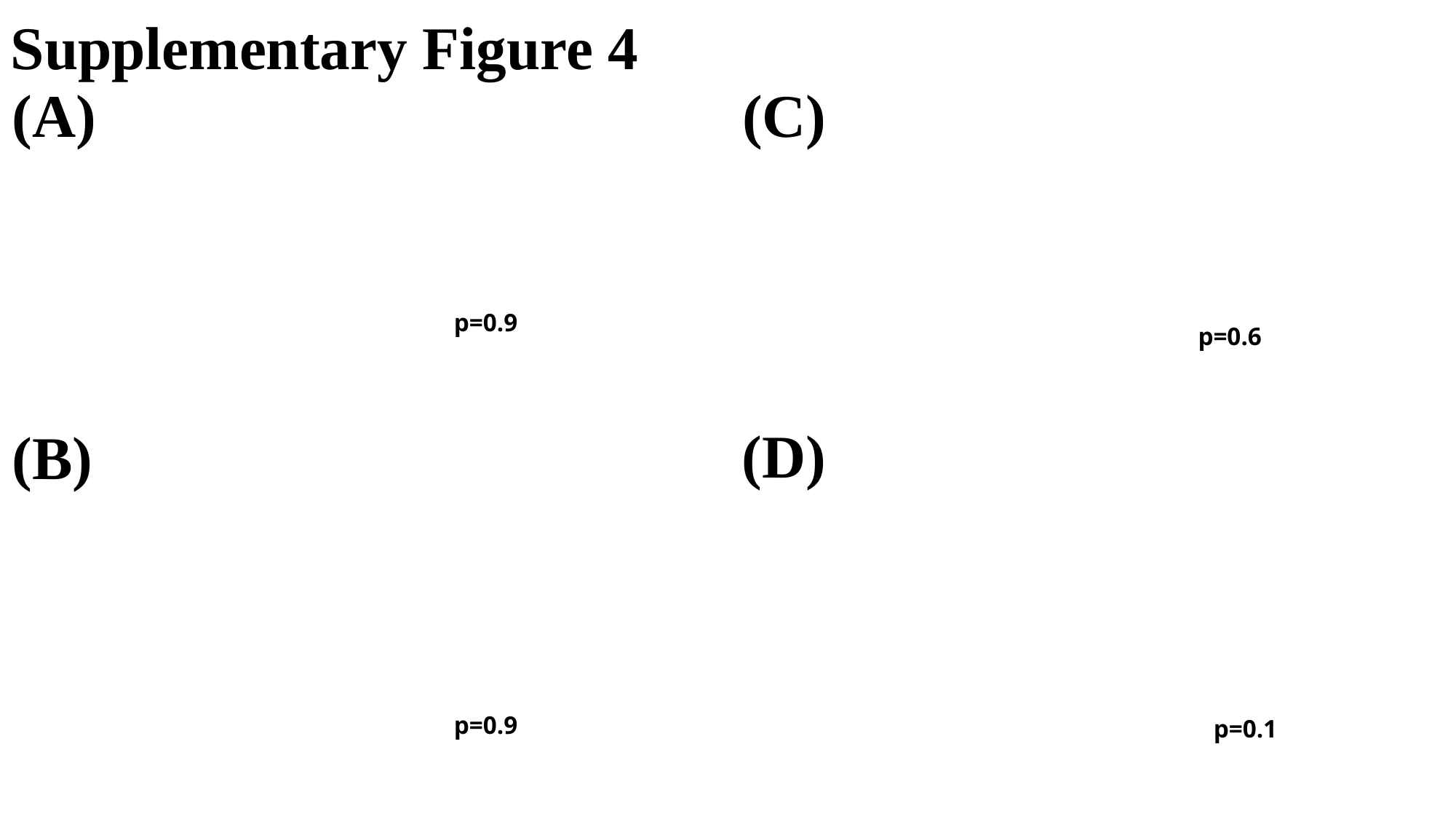

Supplementary Figure 4
(A)
(C)
p=0.9
p=0.6
(D)
(B)
p=0.9
p=0.1

## Slide 6
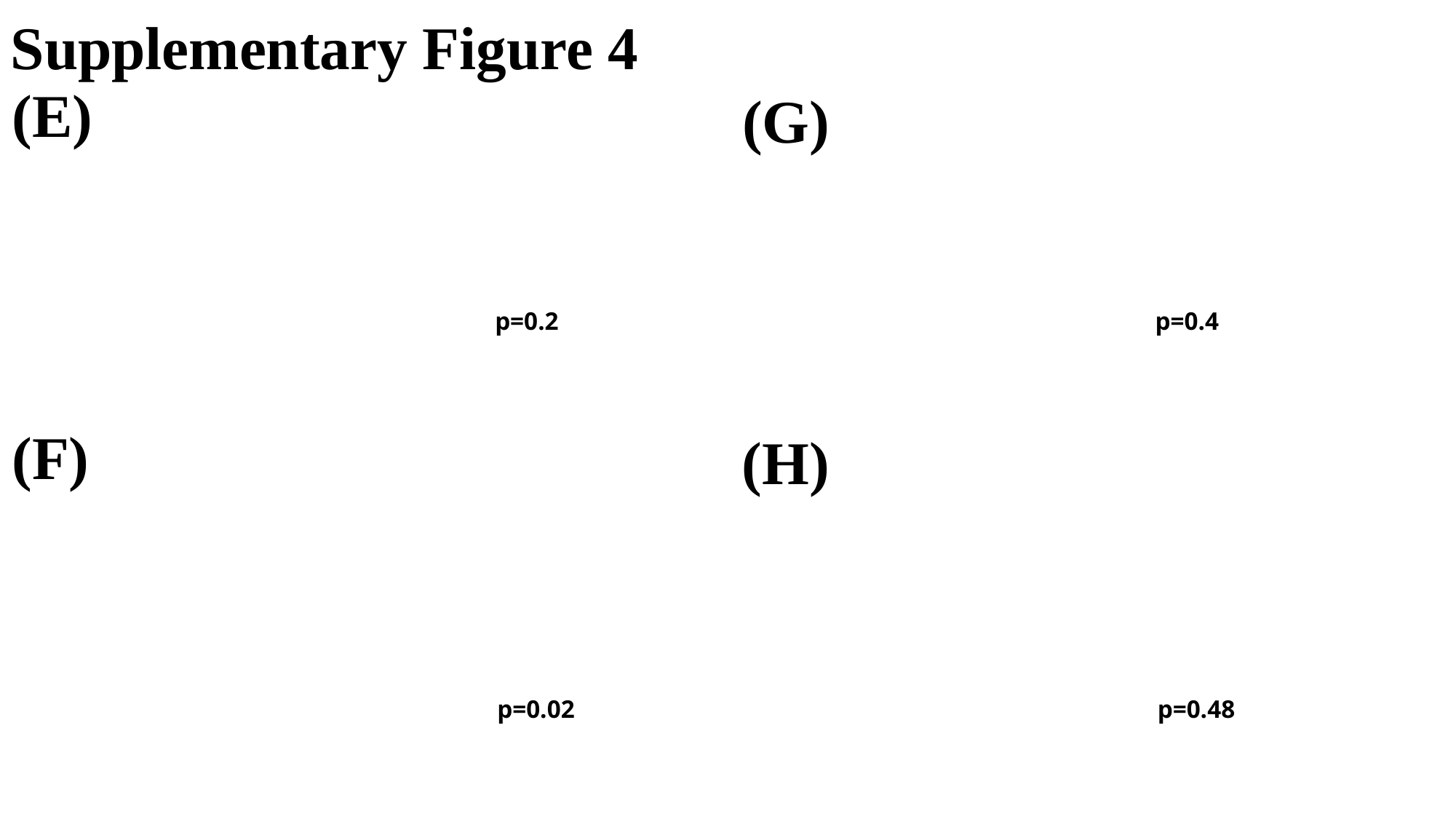

Supplementary Figure 4
(E)
(G)
p=0.2
p=0.4
(F)
(H)
p=0.02
p=0.48
